# Effects of Dietary Isoleucine Supplementation on the Production Performance, Health Status and Cecal Microbiota of Arbor Acre Broiler Chickens

**DOI:** 10.3390/microorganisms11020236

**Published:** 2023-01-17

**Authors:** Hengchen Liu, Jianli Sun, Shuzhen Jiang, Ning Jiao, Libo Huang, Xuejun Yuan, Qinglin Guan, Yang Li, Weiren Yang

**Affiliations:** 1 Key Laboratory of Efficient Utilization of Non-grain Feed Resources (Co-Construction by Ministry and Province), Ministry of Agriculture and Rural Affairs, Department of Animal Science and Veterinary Medicine, Shandong Agricultural University, Tai’an 271018, China; 2 Animal Husbandry Development Service Center of Guangrao County, Dongying 257300, China; 3 College of Life Sciences, Shandong Agricultural University, Daizong Street 61#, Tai’an 271018, China; 4 Shandong Landoff Biotechnology Co., Ltd., Tai’an 271000, China

**Keywords:** broiler chickens, isoleucine, production performance, healthy status, high-throughput sequencing

## Abstract

A total of 24,000 healthy 1-day-old Arbor Acres broilers with similar initial weights were used in this study and fed a basal diet supplemented with 0, 400 and 800 mg/kg isoleucine (Ile), denoted CON, ILE400 and ILE800, respectively. Results revealed that the final body weight, average daily weight gain, and eviscerated carcass rate, of broiler chickens in the ILE400 group were significantly higher than in other groups (*p* < 0.05). In addition, the ILE400 and ILE800 groups had a lower feed conversion rate and a higher survival rate and breast muscle rate (*p* < 0.05), while the abdominal fat rate was significantly lower than the CON group (*p* < 0.05). There were significantly lower serum concentrations of UREA, glucose (GLU) and total cholesterol (TCHO) in the ILE400 and ILE800 groups than in the CON group (*p* < 0.05); glutathione peroxidase (GSH-Px) activity was significantly higher in the ILE400 group than in the other groups, and tumor necrosis factor-alpha (TNF-α) concentration was considerably lower than in other groups (*p* < 0.05). Moreover, interleukin (IL)-10 concentration in the ILE800 group was significantly higher than in the other groups (*p* < 0.05). The ILE400 group significantly down-regulated the mRNA expressions of fatty-acid synthase (*FASN*) and solid alcohol regulatory element binding protein 1c (*SREBP1c*), and significantly up-regulated the mRNA expressions of adipose triglyceride lipase (*ATGL*), hormone-sensitive lipase (*HSL*), lipoprotein lipase (*LPL*) and sirtuin1 (*Sirt1*) (*p* < 0.05). The ILE400 group had significantly higher intestinal villus height than the CON and ILE800 groups, while the ILE800 group had significantly lower intestinal villus height/crypt depth (*p* < 0.05). Furthermore, high-throughput sequencing showed that the Shannon index, and *Verrucomicrobiota*, *Colidextribacter* and *Bacteroides* abundances were significantly higher in the ILE400 group than in the CON group (*p* < 0.05). Interestingly, the ILE800 group reduced the Simpson index, phylum *Firmicutes* and *Bacteroidota* abundances (including genera *Colidextribacter, Butyricicoccus*, *[Ruminococcus]_torques_group, Bacteroides, Alistipes*, *Barnesiella* and *Butyricimonas*), and increased *Proteobacteria* and *Cyanobacteria* (including genera *Dyella*, *Devosia*, *unidentified_Chloroplast* and *Hyphomicrobium*) (*p* < 0.05). Overall, our study showed that adding 400 mg/kg Ile to the diet (diets total Ile levels at 1.01%, 0.90% and 0.87% during the starter, grower and finisher phases, respectively) increased production performance and improved the health status in broiler chickens.

## 1. Introduction

The commercial broiler dominates modern intensive farming with its fast growth, excellent carcass traits and high feed conversion rate [1]. Due to the physiological characteristics of the broilers, there is a high protein demand in the diet. As an essential component of active compounds in the body, the protein has a positive role in promoting the development and health of broilers [2]. With the cost of raw materials increasing, synthetic amino acids such as L-lysine, DL-methionine and L-threonine have been widely used in feed to improve broiler production performance and meat quality and facilitate the production of immunologically active substances [2,3,4,5].

As a functional branched amino acid, isoleucine (Ile) plays an outstanding role in promoting animal growth and development and protecting animal health. Isoleucine may be the third or fourth limiting amino acid in the commercial diet, and the optimal Ile/Lys ratio might affect broiler production performance [6]. The ratio of Ile to Lys as a method of balancing protein in the diet is to optimize protein utilization from the diet [7]. Previous studies have shown that the optimal digestible Ile/Lys in commercial broilers was 0.71 [8,9]. Zhao et al. [10,11] reported that dietary Ile could improve growth performance and the digestibility of nutrients, and enhance intestinal health by modulating intestinal immunity, antioxidant capacity, tight junction proteins and microbial populations. Previous studies in mice demonstrated that Ile supplementation improved immune organ development, immune cell activation and immunoreactive substances secretion, and alleviated intestinal damage developmental delay in mice with colitis [12,13]. The improved production performance of poultry is inextricably linked to normal immune system development, an integrated intestinal barrier, and balanced intestinal microbiota [14,15]. Interactions between complex and diverse microbial communities significantly affect the host’s physiological, immunological and nutritional status, and this complex mechanism potentially has beneficial or detrimental effects on poultry production performance and health [16,17].

Previous studies have focused on Ile requirements, and Ile has mainly been added to diets low in Ile. Recent study has shown that supplementing Ile and Val in a low protein diet improves piglet growth by facilitating nutrient digestibility and intestinal microbiota [18]. To our best knowledge, few studies have been conducted that evaluate the effects of adding Ile alone to the regular diet on production performance, health status and cecal microbiota in broiler chickens. Miranda et al. [19] showed that adding L-Ile to the broilers’ diet improved production performance. This suggests that Ile supplementation in broiler diets is practicable. Therefore, the purpose of this study is to evaluate the effects of Ile on the production performance and health of broilers by dietary Ile levels, and to provide guidance in the usage of Ile in the broiler industry.

## 2. Materials and Methods

### 2.1. Ethical Approval

The Animal Care and Use Committee of Shandong Agriculture University (protocol code SDAUA-2021-019) approved this study.

### 2.2. Animals and Diets

Our study was conducted on a modern intensive broiler breeding farm. A total of 24,000 1-day-old male Arbor Acres broiler chicks, with an average body weight of 45.48 ± 1.35 g, were obtained from a commercial hatchery and were randomly allocated to three groups [8 replicates (pens) per group and 1000 broilers per replicate] for a 42-day study. All broiler chickens were reared in fully automatic standardized chicken coops. As shown in Table 1, three kinds of different basal diets were fed to broiler chicks at the start (day 1–14), growth (day 14–21) and finishing stage (day 21–fencing). The basal diets were based on the nutritional needs of commercial broilers and respected or exceeded the nutrient requirements recommended by the Chinese Ministry of Agriculture (2004) for broilers at different growth periods. The amino acid measurement values and Ile/Lys ratio of the basal diets are shown in Table 2. The Ile (purity, 98.5%) was purchased from CJ (Shanghai, China) Trading Co., Ltd. Three experimental groups were as follows: the control group, broilers fed the basal diet; the ILE400 group, broilers fed the basal diet supplemented with 400 mg/kg Ile; the ILE800 group, broilers fed the basal diet supplemented with 800 mg/kg Ile. The broiler chickens had free access to feed and water and were vaccinated according to the routine immunization schedule throughout the trial. The indoor temperature was set to 31–32 °C 7 days before the trial. It was then decreased by 2 °C per week until the indoor temperature was maintained at 22–23 °C, and the relative humidity was held at 55–60%. Artificial light (10–20 Lux) was provided on a 24 h light program throughout the experimental period.

### 2.3. Sample Collection

At 42 days of age, one broiler chicken with a body weight close to the group was selected from each replicate for sampling. The blood samples were collected from the wing vein and placed in a vacuum collection blood tube (without sodium heparin). The supernatant was aspirated after being centrifuged at 3500× *g* for 10 min at 4 °C, and then stored at −80 °C until analysis. After blood sampling, the broiler chickens were killed by cervical dislocation after CO_2_ asphyxiation. Following dissection of the abdomen, the liver, small intestine and cecum tissues were quickly isolated. Liver and cecal content samples were collected in sterile cryotubes, and stored at −80 °C for further liver qRT-PCR and cecum microbiota analysis. From the middle of the duodenum, an approximately 2 cm section was cut off and repeatedly rinsed with saline, and then immediately fixed in 4% paraformaldehyde for further histological observation.

### 2.4. Serum Biochemistry, Antioxidants and Inflammatory Factors Analysis

The serum concentrations of total protein (TP), albumin (ALB), UREA, glucose (GLU), triglycerides (TG), total cholesterol (TCHO), high-density lipoprotein (HDL) and low-density lipoprotein (LDL) were determined using a fully automated biochemical analyzer (Roche Diagnostic System Inc., Indianapolis, IN, USA). The antioxidant parameters of malondialdehyde (MDA), total superoxide dismutase (T-SOD) and glutathione peroxidase (GSH-Px) in serum were measured using commercial assay kits (Nanjing Jiancheng Bioengineering Institute, Nanjing, China). The serum concentrations of tumor necrosis factor-alpha (TNF-α), interleukin-6 (IL-6) and interleukin-10 (IL-10) were determined by ELISA kits (Jiangsu Meimian Industrial Co., Ltd., Yancheng, China), and the detection steps strictly complied with the kits’ instructions.

### 2.5. Genes Expressions in Liver

The total RNA in each liver sample was extracted using Trizol following the manufacturer’s instructions (Accurate Biology, Changsha, China). The concentrations and purities of total RNA were determined by Spectrophotometer (Denovix DX-11, Wilmington, DE, USA). Then, the cDNA was obtained by reverse transcription kit (Accurate Biology, Changsha, China). The mRNA relative expressions of fatty-acid synthase (*FASN*), peroxisome proliferator activated receptor gamma (*PPAR-γ*), solid alcohol regulatory element binding protein 1c (*SREBP1c*), CCAAT/enhancer binding proteins alpha (*C/EBPα*), adipose triglyceride lipase (*ATGL*), lipoprotein lipase (*LPL*), hormone-sensitive lipase (*HSL*) and sirtuin1 (*Sirt1*) in the liver tissue were determined by using a LightCycler 96 fast real-time PCR system (Roch, Switzerland) and SYBR^®^Green Premix Pro Taq HS qPCR Kit (Accurate Biology, Dalian, China), as previously described by Li et al. [20]. The primer sequences are presented in Table S1. The target gene’s relative mRNA expression was calculated using the threshold cycle ($2^{-\Delta \Delta \mathrm{Ct}}$) method and normalized by the intrinsic reference gene (β-actin) expression.

### 2.6. Duodenal Morphological Analysis

After trimming and washing the samples to remove the intestinal contents, the samples were dehydrated and embedded in paraffin following the usual procedure. Tissue sections were stained with hematoxylin and eosin, and the small intestine morphology was observed and photographed with a Nikon Eclipse 80i (Nikon, Tokyo, Japan) microscope. Villus height (VH) and crypt depth (CD) were measured for each section (Randomly select 10 groups with good morphology) using Image J analysis software, and the VH/CD ratio was calculated.

### 2.7. Cecum Microflora Analysis

The total genomic DNA from broiler chicken cecal contents was extracted by using the CATB method. Then, DNA concentration and purity were measured with 1% agarose gels, and DNA was diluted to 1 ng/μL using sterile water according to the measured concentration. The V3-V4 hypervariable regions of 16S rRNA were amplified using 515F-806R primer [21]. All PCR reactions were performed with 15 μL of Phusion^®^ High Fidelity PCR Master Mix (New England Biolabs, Ipswich, MA, USA). The same volume of 1× loading buffer (containing SYB green) was mixed with PCR products and detected by electrophoresis on a 2% agarose gel. PCR products were mixed in an equal density ratio, and subsequent composite products were purified by using a Qiagen Gel Extraction Kit (Qiagen, Hilden, NRW, Germany). Then, sequencing libraries were constructed using TruSeq^®^ DNA PCR-Free Sample Preparation Kit (Illumina, San Diego, CA, USA) according to the manufacturer’s recommendations. The library quality was measured on the Qubit@ 2.0 Fluorometer (Thermo Scientific, Waltham, MA, USA) and Agilent Bioanalyzer 2100 system. Eventually, the library was sequenced at the Illumina NovaSeq platform and end reads of 250 bp were yielded. A negative control was used in each round of amplification to characterize the sterility of the reagents, and mock bacterial communities were included as controls in the sequencing runs to assess error rates and batch-to-batch variability [22]. After paired-end sequence splicing and quality control, data filtering and chimera removal, the final effective sequences were obtained, which with more than 97% similarity, were clustered into the same OTUs for alpha diversity and beta diversity analysis as previously described in Li et al. [23].

### 2.8. Statistical Analysis

The individual broiler chicken was considered an experimental unit for all variables. All data were evaluated for normal distribution (*W* > 0.05) using the Shapiro–Wilks test. Then, one-way ANOVA was performed using SAS 9.4 (Institute Inc., Cary, NC, USA) statistical software and multiple comparisons were performed using Tukey’s test. Values were expressed as the mean and standard error of the mean (SEM). Probability values of *p* < 0.05 were deemed to be significant differences.

## 3. Results

### 3.1. Production Performance

The effect of dietary supplementation with Ile on production performance is presented in Table 3. The final weight at 42 d and the average daily gain during the experimental period for broiler chickens in the ILE400 group were significantly higher than those in the CON and ILE800 groups (*p* < 0.05). During the testing period, the feed conversion ratio was significantly lower, and the survival rate was significantly higher, in the ILE400 and ILE800 groups (*p* < 0.05). The broiler chickens’ initial body weight and average daily feed intake did not show any significant discrepancy among the three groups (*p* > 0.05). Compared with the CON and ILE800 groups, the ILE400 group had a remarkably increased eviscerated carcass rate (*p* < 0.05). The ILE400 and ILE800 groups had a significantly decreased abdominal fat rate and a significantly increased breast muscle rate compared with the CON group (*p* < 0.05). In addition, there were no remarkable changes among the three groups’ semi-eviscerated carcass rate and thighs muscle rate of the broiler chickens (*p* > 0.05).

### 3.2. Serum Biochemical Parameters

The effect of dietary supplementation with Ile on serum biochemical parameters concentration is shown in Table 4. The concentrations of UREA, GLU and TCHO in the ILE400 and ILE800 groups were remarkably lower than those in the CON group (*p* < 0.05). There were no significant differences in serum TP, ALB, TG, HDL and LDL concentrations among the three groups (*p* > 0.05).

### 3.3. Serum Antioxidant and Inflammatory Parameters

The effects of dietary supplementation with Ile on serum level of antioxidant and inflammatory parameters are displayed in Table 5. The GSH-Px activity in the ILE400 group was markedly higher than that in the CON and ILE800 groups (*p* < 0.05). Compared with the CON group, the ILE400 group had the lowest serum TNF-α concentration (*p* < 0.05), and the ILE800 group had the highest IL-10 concentration among the three groups (*p* < 0.05). No significant changes were observed in serum MDA, IL-6 and T-SOD among the three groups (*p* > 0.05).

### 3.4. Genes Expressions of Liver Fat Metabolism

**Figure 1:**
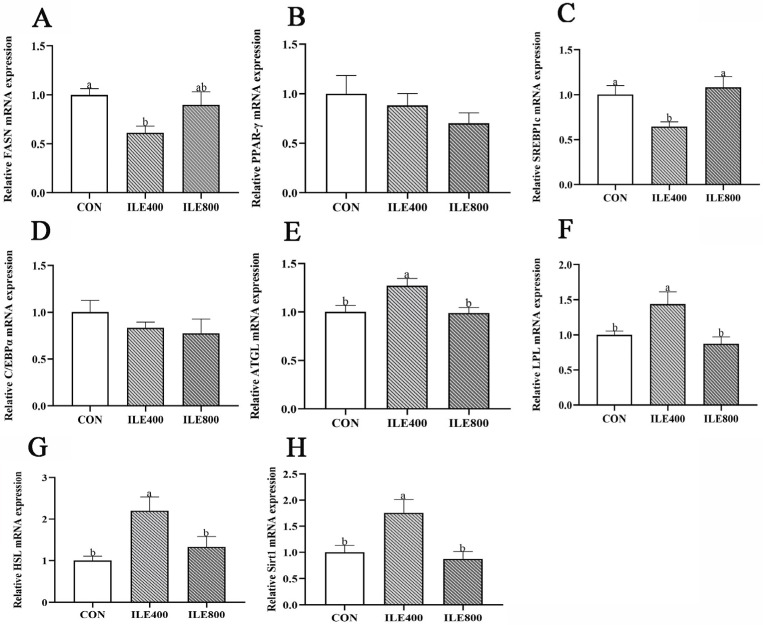
The effect of dietary supplementation with isoleucine (Ile) on the expressions of genes related to liver fat metabolism in broiler chickens. (**A**) *FASN*, fatty-acid synthase; (**B**) *PPAR-γ*, peroxisome proliferator activated receptor-gamma; (**C**) *SREBP1c*, solid alcohol regulatory element binding protein1c; (**D**) *C/EBPα*, CCAAT/enhancer binding proteins alpha; (**E**) *ATGL*, adipose triglyceride lipase; (**F**) *LPL*, lipoprotein lipase; (**G**) *HSL*, hormone-sensitive lipase; (**H**) *Sirt1*, sirtuin1. CON, broiler chickens were fed the basal diet; ILE400, basal diet supplemented with 400 mg/kg Ile; ILE800, basal diet supplemented with 800 mg/kg Ile. The values are presented as the mean and standard error of mean (SEM), and statistically significant differences are those with ^a,b^ *p* < 0.05.

To determine whether Ile affects hepatic lipid metabolism, the expressions of relevant genes were determined in this study. The results indicated that the mRNA expressions of *FASN* (Figure 1A) and *SREBP1c* (Figure 1C) were down-regulated in the ILE400 group compared with the CON and ILE800 groups (*p* < 0.05). Moreover, the ILE400 group showed higher mRNA expressions of *ATGL*, *LPL, HSL* and *Sirt1* (Figure 1E–H) than the CON and ILE800 groups (*p* < 0.05). There were no significant differences between *PPAR-γ* (Figure 1B) and *C/EBPα* (Figure 1D) among the three groups (*p* > 0.05).

### 3.5. Measurements of Duodenal Morphology

**Figure 2:**
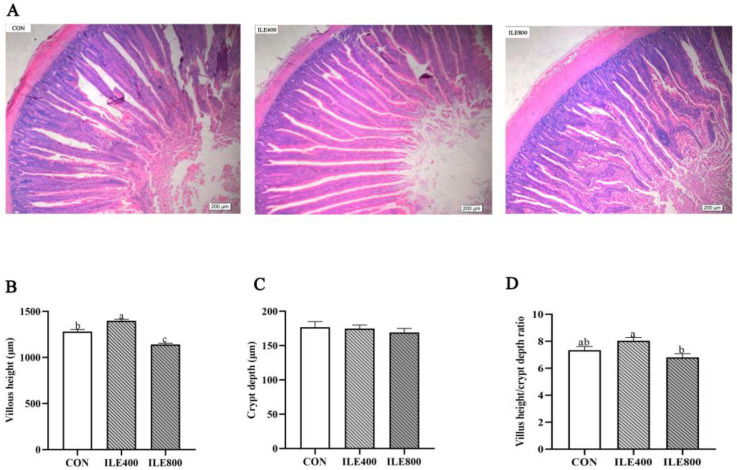
The effect of dietary supplementation with isoleucine (Ile) on duodenal morphology of broilers. (**A**) Hematoxylin and eosin photomicrographs at 40× magnification; (**B**) Villus height; (**C**) Crypt depth; (**D**) Villus height/ Crypt depth ratio. CON, broiler chickens were fed the basal diet; ILE400, basal diet supplemented with 400 mg/kg Ile; ILE800, basal diet supplemented with 800 mg/kg Ile. The values are presented as the mean and standard error of mean (SEM), and statistically significant differences are those with ^a,b,c^ *p* < 0.05.

The effect of supplementation with Ile on duodenal morphology is shown in Figure 2. The analysis of intestinal morphology (Figure 2A) showed that intestinal VH (Figure 2B) was significantly higher in the ILE400 group than in the CON and ILE800 groups (*p* < 0.05), and the VH/CD ratio (Figure 2D) was lower in the ILE800 group (*p* < 0.05). Intestinal CD values (Figure 2C) did not change significantly between the three groups (*p* > 0.05).

### 3.6. Cecal Microbiota Analysis

**Figure 3:**
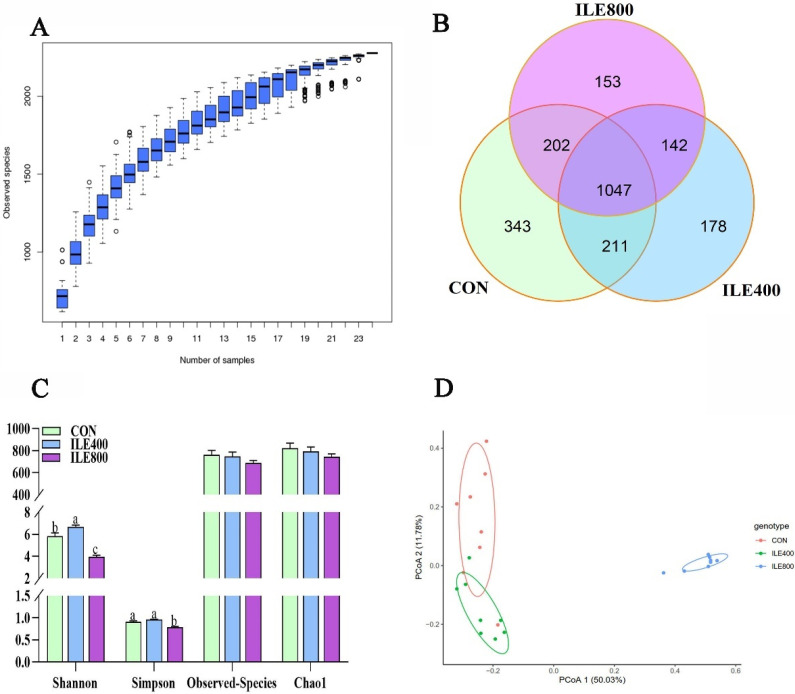
The effect of dietary supplementation with various levels of isoleucine (Ile) on the composition of cecal microbiota. (**A**) The species accumulation boxplot; (**B**) Venn diagram; (**C**) Alpha diversity indexes; (**D**) Principal Coordinate Analysis (PCoA) based on Bray–Curtis distance. CON, broiler chickens were fed the basal diet; ILE400, basal diet supplemented with 400 mg/kg Ile; ILE800, basal diet supplemented with 800 mg/kg Ile. The values are presented as the mean and standard error of mean (SEM), and statistically significant differences are those with ^a,b,c^ *p* < 0.05.

The high-throughput sequencing data is shown in Figure S1. The species accumulation boxplot (Figure 3A) tends to flatten out as the sample size increases up to 24, which indicates that our sample size was sufficient for subsequent analysis and to estimate the species richness of the samples. The Venn diagram (Figure 3B) generated after OTUs clustering with 97% homologous labels of all samples revealed that the CON, ILE400 and ILE800 groups contained 1803, 1578 and 1544 OTUs, respectively. The number of OTUs shared by the three groups amounted to 1047. The results of alpha diversity analysis (Figure 3C) indicated that the Shannon index in the ILE400 group was significantly higher than in the CON and ILE800 groups, while the Simpson index in the ILE800 group was significantly lower than in the CON and ILE400 groups (*p* < 0.05). Regarding the beta diversity of the cecum microbial community, the principal coordinate analysis (PCoA) profile of Bray–Curtis distance (Figure 3D) and the analysis of ANOSIM (Table S2) demonstrated that significant discrepancies existed for each group of the microbial community (*p* < 0.05).

**Figure 4:**
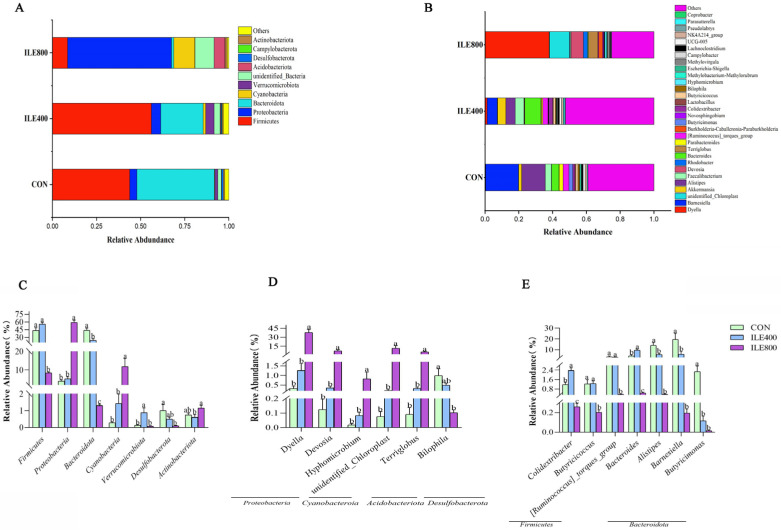
The effect of dietary supplementation with various levels of isoleucine (Ile) on the composition of cecal microbiota. (**A**) Phylum level; (**B**) Genus level; (**C**) The differential microbiota at the phylum level; (**D**,**E**) The differential microbiota at the genus level. CON, broiler chickens were fed the basal diet; ILE400, basal diet supplemented with 400 mg/kg Ile; ILE800, basal diet supplemented with 800 mg/kg Ile. The values are presented as the mean and standard error of mean (SEM), and statistically significant differences are those with ^a,b,c^ *p* < 0.05.

**Figure 5:**
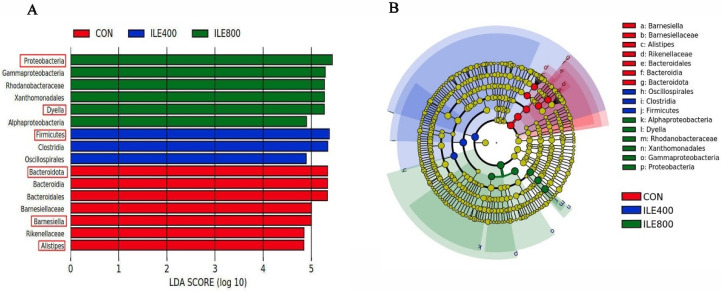
The linear discriminant analysis effect size (LEfSe) analysis of differential microbial taxa in CON, ILE400 and ILE800 groups. (**A**) Distribution histogram of linear discriminant analysis (LDA) values (LDA score > 4.8); (**B**) Evolutionary branching diagram. CON, broiler chickens were fed the basal diet; ILE400, basal diet supplemented with 400 mg/kg Ile; ILE800, basal diet supplemented with 800 mg/kg Ile.

The relative abundances of broiler chickens’ cecal microflora at the phylum level (top10) are shown in Figure 4A. The results indicated that the cecum microbiota in both the CON and ILE400 groups were dominated by the phyla *Firmicutes* (accounting for 43.80% and 56.04%) and *Bacteroidota* (accounting for 43.89% and 24.6%), while the dominant microbiota in the ILE800 group was mainly the phyla *Proteobacteria* (accounting for 58.90%) and *Cyanobacteria* (accounting for 11.94%). Compared with the CON group, the relative abundances of *Firmicutes*, *Bacteroidota* and *Desulfobacterota* (Figure 4C) were remarkably decreased (*p* < 0.05), whereas the relative abundances of *Proteobacteria* and *Cyanobacteria* (Figure 4C) were significantly elevated in the ILE800 group (*p* < 0.05). Compared with the CON group, the relative abundance of *Verrucomicrobiota* was significantly higher in the ILE400 group (*p* < 0.05). The relative abundances of broiler chickens’ cecal microbiota at the genus level (top30) are shown in Figure 4B, and microorganisms with differences at the genus level are shown in Figure 4D,E. The results indicated that the relative abundances of *Dyella*, *Devosi*, *Hyphomicrobium*, *unidentified_Chloroplast* and *Terriglobus* were remarkably increased (*p* < 0.05) and the relative abundances of *Bilophila*, *Butyricicoccus*, *[Ruminococcus]_torques_group*, *Alistipes*, *Barnesiella* and *Butyricimonas* were significantly reduced (*p* < 0.05) in the ILE800 group compared to the CON group. The relative abundances of *Colidextribacter* and *Bacteroides* were significantly increased (*p* < 0.05) in the ILE400 group compared to the CON group. In addition, the LEfSe analysis (Figure 5A,B), which was applied to identify the key microorganisms responsible for the differences among the three groups, showed that *Barnesiella* and *Alistipes* in the CON group, *Closetridia* and *Firmicutes* in the ILE400 group, and *Gammaproteobacteria*, *Aphaproteobacteria* and *Dyella* in ILE800 group, contributed to the differences in cecal microbiota of the three groups.

## 4. Discussion

Isoleucine is commonly considered to be the fourth limiting amino acid in broilers. In our study, dietary supplementation with 400 mg/kg Ile was optimal for broiler body weight, daily gain, and feed conversion. Similarly, previous studies showed that dietary supplementation with Ile could improve the growth performance of poultry, pigs and juvenile Jian carp [10,24,25,26]. Mao [12] showed that dietary supplementation with Ile attenuated the adverse effects of inflammatory bowel disease on growth performance in rats. Moreover, our experiments found that dietary supplementation with Ile could significantly increase the eviscerated carcass rate and breast rate, and reduce abdominal fat. Carcass characteristics represent a significant economic trait. Wise et al. [27] showed that broiler carcass and breast muscle yields increased linearly and quadratically as the Ile to Lys ratio increased in the diet. A recent study also indicated that the broiler abdominal fat rate decreased linearly and the breast rate increased linearly with increasing Ile [28]. Broilers fed on a diet with restricted Ile levels exhibited poorer production performance, but the addition of Ile was able to overcome this disadvantage [29]. The above results showed that adding Ile to the diet could improve the production performance of broiler chickens.

Metabolic health status is a crucial factor contributing to the improved production performance of broilers [30]. In the current study, the serum biochemical indicators, antioxidant parameters and inflammatory cytokines were determined. Serum biochemical indicators reflect the physiological function and metabolic capacity of the organism. In the current study, the supplementation of Ile to the broilers’ diet significantly reduced UREA, GLU and THCO concentrations. UREA could accurately reflect protein metabolism and the balance between amino acids in the body, and its elevated value is usually associated with poultry disease stress conditions [31,32]. The above results suggest that appropriate Ile supplementation could enhance broilers’ amino acid utilization, which may be partly accountable for the broiler’s improved production performance. Isoleucine plays a vital role in improving glucose metabolism and inhabits blood glucose concentration more effectively than leucine [33,34]. A recent experiment in laying hens indicated that serum glucose levels decreased with increasing dietary Ile levels [35]. Doi [34] suggested that Ile regulates blood glucose levels by both stimulating muscle uptake of glucose and increasing systemic glucose oxidation. Ajdar et al. [36] observed that feeding a diet containing Ile and probiotics significantly reduced blood cholesterol concentrations in broiler chickens. Moreover, dietary supplementation with 14.2 g kg^−1^ significantly reduced serum cholesterol, triglyceride and whole-body lipid deposition [37]. These results suggest that Ile can improve blood lipid levels and increase whole body glucose utilization.

Besides, in the current study, dietary supplementation with 400 mg/kg Ile raised serum GSH-Px activity, remarkably. GSH-Px regulates cellular redox capacity by catalyzing the breakdown of hydrogen peroxide and preventing toxic hydroxyl radicals in the body [38]. It has been reported that dietary Ile could enhance antioxidant enzyme activity [11]. Katayama et al. [39] pointed out that Ile protects the intestine from oxidative stress by upregulating GSH content and CAT activity in human intestinal epithelial cells. GSH is an essential non-enzymatic antioxidant in the body, which can efficiently scavenge free radicals and other oxidants under the catalysis of GSH-Px. Gan et al. [40] revealed that the lack or excess of Ile in a grass carp diet leads to increased lipid oxidation and decreased GSH activity in muscles, which is reversed by a moderate level of Ile. These results indicate that Ile enhanced antioxidant capacity in the broiler may be attributed to increased GSH-Px activity. Oxidant stress is often accompanied by inflammatory response [41]. Dietary supplementation with Ile alleviated the negative impact of rotavirus infected piglets by improving immune function [24]. This demonstrated that Ile administration improved animal health during certain challenges [24,42]. In the present study, TNF-α content in the serum of broilers was lowest in the ILE400 group, while IL-10 content in the ILE800 was notably higher than the other two groups. As an essential inflammatory response marker, TNF-α plays a critical role in the immune response by inducing the secretion of other inflammatory molecules and accelerating the inflammatory response [43,44,45]. Multi-effect cytokines, such as IL-10, are essential regulators of immune system stability, exerting anti-inflammatory and immunosuppressive effects [46]. An experiment by Terakura et al. [47] demonstrated that the TNF-α and IL-6 contents in the liver of mice supplemented with 3.0% branched-chain amino acids in the diet were significantly reduced. Another study also showed that dietary supplementation with 1% Ile reduces the inflammatory response in mice with colitis by downregulating inflammation-related cytokine expression through the TLR4/MyD88/NF-κB pathway [12]. The above results suggest that dietary Ile supplementation benefited the enhanced antioxidant capacity and decreased inflammatory response of the broilers.

Disordered gut microbiota can affect the host interaction and relate to local inflammatory occurrence and oxidative stress levels [45,48,49]. In the present study, the ILE800 group had a significantly lower alpha-diversity index than the ILE400 and CON groups. In addition, the beta-diversity results suggested that the intestinal flora composition of the ILE800 group was significantly different from that of the CON and ILE400 groups. At the phylum level, we found that the ILE800 group had significantly lower relative abundances of *Firmicutes*, *Bacteroidetes* and *Desulfobacterota* and significantly higher relative abundances of *Proteobacteria* and *Cyanobacteria* compared to the CON and ILE400 groups. The elevated abundance of *Proteobacteria* leads to dysbiosis and reduced intestinal diversity, increasing the susceptibility to intestinal inflammation and metabolic diseases [50]. At the genus level, we found that the *Dyella* and *Devosia* were significantly elevated in the ILE800 group at 40% and 8%, respectively, and they were probably indicator bacterium for the altered intestinal flora in the ILE800 group. *Dyella* is highly homologous to *Xanthomonadaceae* in *γ-Proteobacteria* [51]. *Devosia* is a type of *Enterobacteriaceae*. Proteins are degraded in the intestine, by gut flora, into branched-chain fatty and potentially toxic products, such as ammonia, amines, phenols and indoles [52,53]. In a high-protein diet, undigested proteins are utilized by intestinal microorganisms through the promotion and proliferation of protein-fermenting bacteria [54]. Research has shown that with increased protein intake, there are increasing number of intestinal bacteria that have a hydrolytic fermentative capacity for proteins, such as *E. coli* [55]. When there is a lack of carbohydrates for energy supply, *E. coli* can preferentially use amino acids as an energy source [56]. Thus, we hypothesize that the rise of undigested protein in the intestine may facilitate the proliferation of bacteria such as *Proteobacteria*. The raised abundance of *Cyanobacteria* has been associated with the occurrence of human diseases [57]. *Firmicutes* and *Bacteroidetes* were related to the production of short-chain fatty-acids such as butyrate, acetate and propionate [58]. In the ILE400 group, the *Colidextribacter* and *Bacteroides* abundances were significantly higher. *Bacteroides* enhance the body’s immunity by interacting with the host immune system [59]. *Colidextribacter* contributes to regulating the inflammatory response and maintains the integrity of the gut mucosa [21]. This indicated that the dietary addition of 400 mg/kg Ile affects the composition of the intestinal flora and increases the abundance of beneficial bacteria. In the ILE800 group, the abundances of *Butyricicoccus*, *Ruminococcus*, *Alistipes, Barnesiella* and *Butyricimonas* were significantly decreased. The *Barnesiella* and *Alistipes* abundances positively correlates with single-chain fatty-acid production [60]. *Butyricimonas* and *Ruminococcus* convert the glucose from food into butyrate, which can provide energy to intestinal cells, promote metabolism, reduce harmful bacteria colonization and protect intestinal health [61]. Furthermore, our study also found that the duodenal VH and VH/CD ratio were significantly lower in the ILE800 group. Villous and crypt structures in the small intestine are primarily responsible for nutrient penetration and intestinal cell proliferation [62]. Higher VH and VH/CD values generally mean better intestinal morphology. Research has shown that a lack or excess of methionine in the diet can reduce the intestinal VH and VH/CD ratio of piglets, and affect intestinal development [63]. This suggests that appropriate Ile levels in the diet contributes to nutrient absorption and intestinal health. Our results indicated that adding 800 mg/kg Ile to the diet reduced the intestinal VH and VH/CD ratio and decreased the cecum’s flora diversity, and increased the abundance of some harmful bacteria.

The current study also showed that the ILE400 group significantly suppressed lipogenic genes expressions, such as *FASN* and *SREBP1c*, and promoted lipolytic gene expressions, such as *ATGL* and *HSL*. The liver is the main organ involved in fat metabolism in poultry, and abdominal fat deposition is a major component of fat metabolism [64]. Similar results were observed in tests on mice and weaned piglets [65,66]. *SREBP1s*, a membrane-bound transcription factor, regulates adipogenesis by enhancing fatty-acid synthesis and accelerating the accumulation of triglycerides [67]. It has been reported that *SREBP1c* can induce the expressions of lipogenic genes, such as *FAS*, *ACC1* and *SCD1* [68]. Triglycerides are hydrolyzed to glycerol and fatty-acids by the action of *ATGL* and *HSL*, which serve as essential rate-limiting enzymes for lipolysis [69]. In addition, Ma [66] suggested that feeding 1.5% Ile in the diet of obese mice reduced adipogenic genes expression and prompted lipid browning. The above results suggest that Ile inhibits lipid synthesis and promotes lipolysis by regulating the effect of genes related to hepatic lipid metabolism. Vianna et al. [70] pointed out that the reduction in fat synthesis by leucine may be linked to the regulation of energy expenditure in the body. We examined the mRNA expression of *Sirt1*, and the results showed that the ILE400 group significantly upregulated the expression of *Sirt1*. It was found that the downregulation of lipogenic genes (*FASN*, *ACC1* and *SREBP1c*) notably decreased after the knockdown of *Sirt1* in fasting conditions [71]. Walker [71] showed that the deacetylation of *SREBPs* by *Sitt1* led to a decrease in their activity, which resulted in the depression of adipogenesis. Interestingly, Zhou et al. [72] indicated that mice fed a leucine-supplemented diet facilitated fat deposition and accelerated fat browning, which is inconsistent with our study. This may be due to the varying Ile levels and animal models. In conclusion, adding 400 mg/kg Ile to the diet can suppress lipid deposition in the liver and benefit fat metabolism.

## 5. Conclusions

In the present study, supplementation with 400 mg/kg Ile (total dietary Ile levels of 1.01%, 0.90% and 0.87% during the starter, grower and finisher phases, respectively) improved the broilers’ production performance and health status, and changed the composition of their intestinal flora.

## Figures and Tables

**Table 1:** Composition and nutrient levels of the basal diets (air-dry basis).

Items	Start Stage 0–14 d	Growth Stage 14–21 d	Finishing Stage 21–42 d
Ingredients, %			
Corn	47.44	49.48	44.83
Soybean meal	35.81	28.43	26.21
Wheat	5	10	15
Fermented sesame meal	2	2	2
Fermented cottonseed protein	3	3	3
Soybean oil	2.33	2.60	4.96
Stone powder	1.42	1.24	1.12
Dicalcium phosphate	0.93	0.78	0.51
Sodium chloride	0.17	0.20	0.12
Liquid methionine	0.37	0.42	0.39
L-lysine HCl	0.52	0.71	0.68
Premix ^1^	1.01	1.14	1.18
Total	100.00	100.00	100.00
Nutritional levels ^2^, %			
Poultry Metabolizable Energy, MJ/Kg	11.97	12.43	13.08
Crude protein	22.4	20.2	19.5
Calcium	0.78	0.68	0.60
Available phosphorus	0.34	0.31	0.26
Chicken available methionine	0.63	0.63	0.60
Chicken available lysine	1.25	1.20	1.14

^1^ Premix is provided per kg of feed: VA, 7500 IU; VD3, 2500 IU; VB1, 0.65 mg; VB2, 7.0 mg; VB6, 1.9 mg; VB12, 11 mg; Pantothenic acid, 11 mg; VE, 8.5 IU; Choline chloride, 750 mg; Cu, 8 mg; Fe, 80 mg; Zn, 40 mg; Mn, 60 mg; Se, 0.15 mg; I, 0.35 mg. ^2^ Nutritional levels were calculated values.

**Table 2:** The contents of amino acids in the basal diets.

Items	Start Stage 0–14 d	Growth Stage 14–21 d	Finishing Stage 21–42 d
Amino acid, %			
Lysine	1.55	1.48	1.46
Isoleucine	0.97	0.86	0.83
Isoleucine/Lysine	0.62	0.58	0.56
Methionine	0.40	0.62	0.31
Threonine	1.04	1.00	0.96
Tryptophan	0.27	0.24	0.22
Leucine	1.84	1.69	1.58
Valine	1.09	1.02	1.00
Arginine	1.44	1.28	1.27
Histidine	0.64	0.61	0.59

**Table 3:** The effect of dietary supplementation with isoleucine (Ile) on broiler production performance.

Items	CON	ILE400	ILE800	SEM	*p* Value
Growth Performance (day 1 to 42)					
Initial Body Wight, g	45.30	45.91	45.24	0.279	0.571
Final Body Weight, g	2737.37 ^b^	2818.57 ^a^	2756.97 ^b^	10.953	0.003
Average Daily Gain, g/d	64.13 ^b^	66.07 ^a^	64.60 ^b^	0.261	0.003
Average Daily Feed Intake, g/d	113.01	109.98	108.20	0.867	0.067
Feed Conversion Ratio	1.74 ^a^	1.66 ^b^	1.67 ^b^	0.011	0.002
Survival Rate, %	93.82 ^b^	96.54 ^a^	96.01 ^a^	0.379	0.003
Carcass Characteristics					
Eviscerated Carcass Rate, %	74.38 ^b^	76.33 ^a^	75.16 ^b^	0.224	<0.001
Semi-Eviscerated Carcass Rate, %	84.18	84.36	84.49	0.081	0.321
Abdominal Fat Rate, %	3.03 ^a^	1.88 ^b^	2.12 ^b^	0.130	<0.001
Breast Muscle Rate, %	25.04 ^b^	26.95 ^a^	27.12 ^a^	0.292	0.002
Thighs Muscle Rate, %	17.24	17.14	17.13	0.298	0.986

The values are presented as the mean and standard error of mean (SEM). Statistically significant differences are those with ^a,b^ *p* < 0.05. CON, broiler chickens receiving basal diet; ILE400, broiler chickens receiving basal diet supplemented with 400 mg/kg Ile; ILE800, broiler chickens receiving basal diet supplemented with 800 mg/kg Ile.

**Table 4:** The effect of dietary supplementation with isoleucine (Ile) on the serum biochemistry of broiler chickens.

Items	CON	ILE400	ILE800	SEM	*p* Value
TP, g/L	21.26	22.21	22.76	0.652	0.664
ALB, g/L	6.70	6.86	6.26	0.222	0.665
UREA, mmol/L	0.49 ^a^	0.28 ^b^	0.32 ^b^	0.021	<0.001
TG, mmol/L	0.27	0.24	0.24	0.010	0.483
GLU, mmol/L	11.02 ^a^	8.55 ^b^	8.03 ^b^	0.330	<0.001
TCHO, mmol/L	3.25 ^a^	2.42 ^b^	2.49 ^b^	0.110	<0.001
HDL, mmol/L	0.99	1.02	0.93	0.041	0.622
HDL, mmol/L	0.50	0.54	0.45	0.030	0.452

The values are presented as the mean and standard error of mean (SEM). Statistically significant differences are those with ^a,b^ *p* < 0.05. CON, broiler chickens receiving basal diet; ILE400, broiler chickens receiving basal diet supplemented with 400 mg/kg Ile; ILE800, broiler chickens receiving basal diet supplemented with 800 mg/kg Ile.

**Table 5:** The effects of dietary supplementation with isoleucine (Ile) on the serum antioxidants and inflammatory factors of broiler chickens.

Items	CON	ILE400	ILE800	SEM	*p* Value
MDA, nmol/L	2.65	2.35	2.61	0.064	0.099
T-SOD, U/mL	73.52	82.08	82.51	1.782	0.057
GSH-PX, U/mL	1583.87 ^b^	1763.87 ^a^	1608.39 ^b^	24.307	<0.001
TNF-α, ng/L	74.38 ^a^	59.37 ^c^	66.40 ^b^	1.925	0.001
IL-6, ng/L	55.61	54.34	51.32	1.928	0.675
IL-10, ng/L	33.48 ^b^	31.98 ^b^	37.65 ^a^	0.714	<0.001

The values are presented as the mean and standard error of mean (SEM). Statistically significant differences are those with ^a,b,c^ *p* < 0.05. CON, broiler chickens receiving basal diet; ILE400, broiler chickens receiving basal diet supplemented with 400 mg/kg Ile; ILE800, broiler chickens receiving basal diet supplemented with 800 mg/kg Ile.

## Data Availability

All sequencing data are stored in the sequence read archive of the National Center for Biotechnology Information under the accession number PRJNA905560.

## Supplementary Materials

The following supporting information can be downloaded at: https://www.mdpi.com/article/10.3390/microorganisms11020236/s1, Table S1: Primer sequences used for quantitative real-time PCR; Table S2: Analysis of differences in ANOISM among the groups; Figure S1: Sequencing data.
