# Supplementary material for: Effects of Dietary Isoleucine Supplementation on the Production Performance, Health Status and Cecal Microbiota of Arbor Acre Broiler Chickens
Source: Microorganisms. 2023 Jan 17;11(2):236. doi: 10.3390/microorganisms11020236 (PMC9958568; doi:10.3390/microorganisms11020236)
Supplement: Supplementary file 1 [file microorganisms-11-00236-s001.zip › microorganisms-2092291-supplementary.pdf]

Table S1 Primer sequences used for quantitative real-time PCR

Gene	GenBank	Primer sequences [a]	size, bp
β-actin	NM_205518.1	F: ATTGTCCACCGCAAATGCTTC	113
		R: AAATAAAGCCATGCCAATCTCGTC	
Sirt1	NM_001004767.1	F: CACGCCTTGCTGTAGACTTCC	148
		R: ATGAACTTGTGGCAGAGAGATGG	
LPL	NM_205155.3	F: CAGTGCAACTTCAACCATACCA	150
		R: AACCAGCCAGTCCACAACAA	
FASN	NM_205155.3	F: TCCTGACTCTCCTGACCATTACT	126
		R: TGTTCCATGACCATGCCAAGA	
ATGL	NM_001113291.1	F: GCAACCTCTACCGCCTCTCAA	132
		R: TTGGACGCTGAAGGTGAAGGA	
PPAR-γ	373928	F: GTGACCTTAATTGTCGCATCCAT	135
		R: GCTTCTCCTTCTCCGCTTGTG	
HSL	112533458	F: CCATCCTGTCCGTCGATTACTC	80
		R: GCAGTAGGCGTAGAAGCACTC	
SREBP1	NM_204126.3	F: CGCTACCGCTCATCCATCAAC	93
		R: CCTCAGGATCGCCGACTTGTT	
C/EBPα	NM_001031459.2	F: CGCTACCGCTCATCCATCAAC	99
		R: CCTCAGGATCGCCGACTTGTT	

[a] F, forward; R, reverse

Table S2 Analysis of differences in ANOISM among the groups

Treatment	[1]*r* value	[2]*p* value
CON *vs* ILE400	0.433	0.001
CON *vs* ILE800	1	0.001
ILE400 *vs* ILE800	1	0.001

[1] r value between (-1,1); r value > 0 indicating significant differences between groups, r value < 0 indicating within difference are greater than inter-group differences. [2] $p < 0.05$ indicates statistical significance. CON, broiler chickens receiving basal diet; ILE400, basal diet supplemented with 400 mg/kg Ile; ILE800, basal diet supplemented with 800 mg/kg Ile

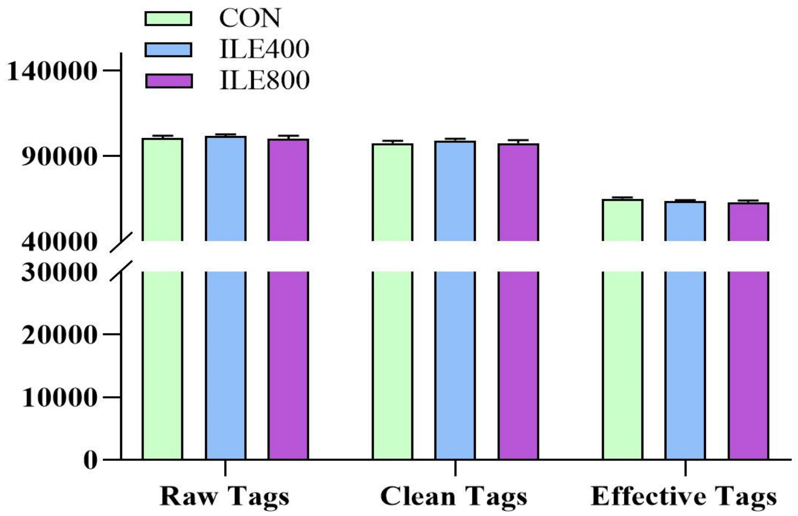

Figure S1. Sequencing data; Raw Tags indicate the raw sequence; low quality and short length sequences in raw tags are filtered out to get Clean Tags and Effective Tags are the final sequence for subsequent analysis. CON, broiler chickens were fed the basal diet; ILE400, basal diet supplemented with 400 mg/kg Ile; ILE800, basal diet supplemented with 800 mg/kg Ile. The values are presented as the mean and standard error of mean (SEM), and statistically significant differences were those with $^{a\,b}$ $p < 0.05$.
